# Elimination of HIV transmission in Japanese MSM with combination interventions

**DOI:** 10.1016/j.lanwpc.2022.100467

**Published:** 2022-05-10

**Authors:** Yijing Wang, Junko Tanuma, Jinghua Li, Kota Iwahashi, Liping Peng, Chun Chen, Yuantao Hao, Stuart Gilmour

**Affiliations:** a School of Public Health, Sun Yat-sen University, Guangzhou, China; b Graduate School of Public Health, St. Luke's International University, Tokyo, Japan; c AIDS Clinical Center, National Center for Global Health and Medicine, Tokyo, Japan; d Community Center Akta, Tokyo, Japan; e Department of Mechanical and Automation Engineering, The Chinese University of Hong Kong, Hong Kong Special Administrative Region, China; f Shenzhen Research Institute, The Chinese University of Hong Kong, Shenzhen, China; g Peking University Center for Public Health and Epidemic Preparedness & Response, Peking University, Beijing, China; h Sun Yat-Sen Global health Institute and the center for Health Information Research, Sun Yat-Sen University, Guangzhou, China

**Keywords:** HIV infections, Japan, Incidence, Policy, Interventions, Mathematical models

## Abstract

**Background:**

Japan has a concentrated HIV epidemic, with the majority of transmission among men who have sex with men (MSM). This study aimed to explore the effect of behavioral and biomedical interventions on the HIV epidemic and forecast the time required to eliminate HIV transmission among Japanese MSM.

**Methods:**

A deterministic compartmental model was built to estimate and forecast the HIV epidemic among Japanese MSM from 2010 to 2050. Elimination of HIV transmission among MSM was defined as incidence less than 1/1000 person-years. The time required for eliminating HIV transmission under different scenarios was calculated.

**Findings:**

Under the current policies, HIV transmission cannot be eliminated by 2050. Both behavioral and biomedical interventions can achieve elimination of HIV among MSM by 2050 with annual number of sexual partners among high-risk MSM less than 9, or with condom use rate above 65%, or with testing and treatment rate above 80%, or with more than 10% PrEP coverage rate. Under comprehensive interventions, HIV elimination will be achieved in 2032, 2025 and 2024 using weak, moderate and strong intervention combinations, respectively.

**Interpretation:**

Both behavioural and biomedical interventions can achieve elimination of HIV among MSM by 2050, but comprehensive interventions can accelerate the realization of this goal with higher feasibility.

**Funding:**

This study was funded by a Health and Labor Sciences Research Grant (Research on HIV/AIDS) from The Ministry of Health Labour and Welfare (21HB0701) and National Natural Science Foundation of China (No. 81773543 and 81973150), and the KC Wong Education Foundation.

**Research in context**

**Evidence before the study**

We searched pubmed for studies related to HIV in Japan, HIV elimination in Japan, and HIV policy. We also performed a Japanese language websearch, and searched the available policy documents in Japanese for information about the current progress towards HIV elimination in Japan. Past studies showed that the incidence of HIV may have plateaued in the last 5 years, but past mathematical modeling studies showed prevalence will continue to grow without additional policy interventions, in particular the expansion of voluntary testing and counseling (VCT) combined with pre-exposure prophylaxis (PrEP) and improved entry into treatment. No past studies identified conditions for or timelines to achieve elimination.

**Added value of the study**

Although past studies have identified likely future trends in HIV incidence and prevalence, this is the first study to assess conditions for the elimination of HIV in Japan. This study uses an up-to-date mathematical model to assess improvements in behavioral and biomedical strategies required to achieve elimination of HIV. It describes a viable and feasible pathway to achieve elimination based on current understanding of the HIV epidemic in Japan, and gives an approximate estimate of how long it will take to achieve elimination.

**Implications of all the available evidence**

Although incidence may have plateaued in Japan, prevalence will continue to rise under current policies and the epidemic will not be stopped under the status quo. However, a combination of small reductions in behavioral risk, combined with moderate improvements in testing and speed of treatment entry, would be sufficient to eliminate new cases of HIV by the middle of the next decade at the latest.

Alt-text: Unlabelled box

## Introduction

The HIV epidemic has been a major global health challenge since the early 1980s^1^ with 32.7 million cumulative deaths from AIDS and 38.0 million people living with HIV (PLWH) at the end of 2019.^2^ Great efforts have been made globally to prevent HIV transmission, but the pandemic persists. In the early years of the pandemic, condom use was the only method to prevent HIV transmission. After effective antiretroviral therapy (ART) was found to prevent HIV transmission to sexual partners,^3^ treatment as prevention (TasP) and test-and-treat^4^ became major components of global HIV prevention strategies ^3^^,^^5^ and led to the Fast Track strategy of the Joint United Nations Programme on HIV/AIDS (UNAIDS) to end the AIDS pandemic by 2030,^6^ along with the associated 2025 targets^7^ for interventions required to achieve this goal.

In Japan, there has been a concentrated HIV epidemic among men who have sex with men (MSM) and MSM are considered the key population. The number of new HIV notifications among Japanese MSM has outpaced those of all the other routes since the early 2000s.^8^ While the MSM population accounts for 2.9% to 4.6% of the Japanese male population,^9^, ^10^, ^11^ it disproportionately accounted for 75.4% of male HIV cases, 58.1% of male AIDS cases, and 66% of new HIV notifications in 2018,^12^ which is much higher than those in other Asian countries.^13^ Therefore, the key to the goal of ending the HIV epidemic in Japan lies in controlling HIV transmissions among MSM.

Japan has established a nationwide scheme for free and anonymous testing at public health centers. However, the HIV testing rate remains low among the key population, leading to high rates of late diagnosis of HIV infection. Among newly diagnosed HIV cases in 2018, 28.6% were in the stage of AIDS.^14^ The 2017 Love Life and Sexual Health (LASH) survey of treatment and prevention intentions targeting the MSM population found 62% of respondents had ever had an HIV test and 55.4% of them had their test within a year,^15^ showing the testing rate was far from the recommendation of annual HIV testing to MSM in the guidelines of many other countries.^16^^,^^17^

Japanese ART guidelines are consistent with those of other countries in recommending immediate ART after HIV diagnosis, but there have been challenges to implementing a comprehensive test-and-treat strategy. Although 70% of the medical cost can be covered by health insurance, the remaining cost for ART is still a large financial burden for PLWH that increases the difficulty of payment and acts as a barrier to early entry to HIV treatment. In recognition of this, there has been a disability certificate policy for PLWH^18^ which reduces the co-payment to less than JPY 20,000 per month.^19^ However, there are clinical indications that limit issuing the disability certificate in levels of CD4 count and HIV viral loads and AIDS history^20^^,^^21^ and the process of issuing the disability certificate usually takes two months for asymptomatic patients. Those conditions have prevented Japan from achieving a full test-and-treat strategy.

Pre-exposure prophylaxis (PrEP) is another core element of the current global HIV prevention strategies,^22^, ^23^, ^24^, ^25^ though, Japan has not officially approved the usage of antiretroviral drugs for PrEP. It is legally possible to purchase antiretroviral drugs for PrEP online if the purchaser is able to bear the out of pocket costs.^26^ In the 2017 LASH report, 63.1% of respondents indicated willingness to take anti-HIV drugs (PrEP) to prevent HIV infection, while their top concern in using PrEP was its cost.^15^

Traditional venue-based (such as bars, clubs or saunas) and online-based activities led by non-governmental organizations (NGOs) have taken important roles in HIV prevention campaigns that encourage behavioral changes among key populations. Although the Japan Foundation for AIDS Prevention (JFAP), a non-governmental foundation, redistribute funds and donations to NGOs to support their HIV prevention activities in the community-based approach,^18^^,^^27^ government support is not enough to effectively reach out to key populations and change their behavior.^28^^,^^29^ It is therefore essential to identify to what extent behavioral and biomedical interventions need to be implemented to plan effective HIV prevention programs among Japanese MSM. We previously published estimated trends in HIV prevalence among Japanese MSM under the enhanced test-and-treat strategy and PrEP. However, little research so far has identified the collected impact of behavioral and biomedical interventions in Japan. In this study, we explored the effects of the combination of partner reduction, increasing condom use, enhancing test-and-treat, and introducing PrEP on the future HIV epidemic among Japanese MSM and project the time required to eliminate HIV transmission by mathematical modelling.

## Methods

### A deterministic compartmental model of HIV

A deterministic compartmental model was built to reflect the mechanism of HIV progression,^23^ running on the Japanese MSM population aged 18-59 years old. This population was further divided into low risk MSM (LRMSM) and high risk MSM (HRMSM) groups based on the number of sexual partners they had in the past year. LRMSM were defined as all men up to the 80th percentile of partner numbers, and HRMSM were the men in the highest quintile of partner numbers. The model was applied to both LRMSM and HRMSM.

**Figure 1:**
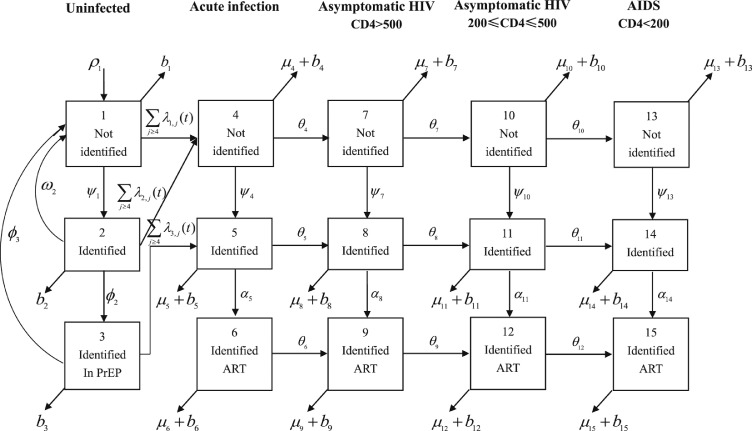
Compartmental structure of the model.

The model divides the MSM population into 15 compartments based on their HIV serostatus, CD4 count, recognition of HIV serostatus and treatment activity. The structure of the model is shown in Figure 1. Five columns represent one uninfected stage and four different progressive stages of HIV infection. Acute infection is the earliest stage of HIV infection, in which the virus is multiplying rapidly so the newly infected person will be highly infectious during this time, followed by three stages of HIV and AIDS. Three rows represent recognition of HIV serostatus and treatment engagement. From top to bottom, these are untested, tested but not on treatment, and on treatment, respectively. For the first column in the last row, “treatment” represents PrEP. The rate of change of the number of people in each compartment is described by an ordinary differential equation (ODE). Table 1 shows the parameters involved in the model. A detailed description of the system of ODEs is given in Supplementary Section 1.

**Table 1:** Model parameters

Parameter	Value	References
*Demographic*		
Initial population (age 18-59)		
Men	35763734	
MSM (%)	3.5	9, 10, 11
Low-risk MSM (%)	80	^15^
High-risk MSM (%)	20	^15^
Background maturation and entry rates		
Annual maturation rate, male	0.0271	Calculated^a^^22^
Annual entry rate, male	0.0167	Calculated^b^^22^
Background mortality rate		
Annual mortality rate, male	0.00486	^22^
Annual mortality rate without ART		
Acute	0.003	^46^
Asymptomatic (CD>500)	0.03	^47^
Asymptomatic (200<=CD4<=500)	0.1	^48^
AIDS (CD4<200)	0.48	^48^
Annual mortality rate with ART		
Acute	0.002	^46^^,^^49^
Asymptomatic (CD>500)	0.002	^46^^,^^49^
Asymptomatic (200<=CD4<=500)	0.01	^46^^,^^49^
AIDS (CD4<200)	0.02	^46^^,^^49^
*Biological*		
Duration of HIV progression status converted to months*		
Acute to CD4>500	3	^50^
CD4>500 to 200<=CD4<=500	14.3	^50^
200<=CD4<=500 to CD4<200	80.33	^50^
Probability of HIV transmission per partnership		
Acute (within 3 months)	0.21	^23^
Asymptomatic (CD>500)	0.003	^23^
Asymptomatic (200<=CD4<=500)	0.045	^23^
AIDS (CD4<200)	0.12	^23^
Reduction in infectivity (multiplicative) due to ART	0.99	^38^
*Behavioral*		
Annual number of partners		
MSM, total	4.1	^15^
Low risk MSM	1.6	Calculated^c^^15^
High risk MSM	14.1	^15^
Condom use (% of sexual encounters)		
Condom use rate (%)	35%	^29^^,^^51^
Condom effectiveness	0.9	^52^
Others		
Proportion of members of one group having sexual interaction with members of the other group	0.3	Assumed
Reduction in sexual behavior after HIV diagnosis	0.2	Assumed
Reduction in sexual behavior among AIDS patients	0.9	Assumed
*Biomedical*		
HIV testing rate		
Proportion of population tested in past 12 months, %	35%	^10^^,^^15^
Rate of detection of HIV through passive case-finding	0.1	Assumed
Rate of detection of AIDS through passive case-finding	1	Assumed
Average duration that uninfected individuals remain identified after testing in risk	12 months	
Monthly entry rate to ART (treatment rate)		
Acute	0.2	Calculated^d^
Asymptomatic (CD>500)	0.29	Calculated^d^
Asymptomatic (200<=CD4<=500)	0.38	Calculated^d^
AIDS (CD4<200)	0.43	Calculated^d^
Pre-exposure prophylaxis		
Rate of uninfected people start taking PrEP (PrEP coverage rate)	-	Based on the scenarios
Rate of PrEP dropout	0	100% adherence
PrEP Effectiveness	0.9 under 100% adherence	^30^
*Transmission force*		
Transmission force^e^ (k=1,2 means not in PrEP, k=3 means in PrEP)	-	^23^

NOTE: *Duration doubled after ART. Calculated^a^: The rate of aging. Calculated^b^: The rate of entering the 18-59 age group. Calculated^c^: Annual number of partners in LRMSM was calculated coordinated with the proportion of people in each group and the annual number of partners in HRMSM to ensure the annual number of partners of 4.1 in the whole population. Calculated^d^: Obtained using survival analysis of the data obtained from the clinic cohort at AIDS Clinical Center. Transmission force^e^: Transmission force represents the rate of uninfected people entering the infected population, which is the sum of force of infections with each infectious compartment. The detailed calculations are shown in Supplementary Section 1.2 and 1.3.

### Ethical considerations

This is a mathematical modeling study with no experiments involving humans, so ethical considerations are not applicable. The analysis was conducted in 2020 – 2021 using data collected from official sources over that time period and forecast to 2050.

### Intervention scenarios

We changed the values of the number of sexual partners, condom use rate, testing and treatment rate and PrEP coverage rate in the compartmental model from 2022 to model enhancing interventions from 2022, and then explored the effects of single measure behavioral or biomedical intervention and comprehensive combination of those interventions. The specific scenarios are as follows.

• Scenario 0 (Status Quo): No additional intervention over the status quo of current policies. The information under status quo is shown in Table 1.

#### Behavioral interventions

• Scenario 1 (Partner reduction): Control the annual number of sexual partners per capita of HRMSM no more than 14 (i.e., between 1 to 14), other conditions maintain the status quo level.
• Scenario 2 (Increased condom use rate): Increase the overall condom use rate to over 40% (i.e., between 40% to 100%), with 90% condom effectiveness, other conditions maintain the status quo.
• Scenario 3 (Behavioral combination intervention): Control the annual number of sexual partners per capita of HRMSM to no more than 14, increase the overall condom use rate to over 40%, other conditions maintain the status quo.

#### Biomedical interventions

• Scenario 4 (Enhanced test-and-treat): Increase both the overall testing rate and the treatment rate to over 50% (i.e., between 50% to 100%), representing TasP and the test-and-treat strategies, other conditions maintain the status quo.
• Scenario 5 (Introducing PrEP): Introduce PrEP to both LRMSM and HRMSM, with coverage rates between 10% to 100%, with 90% effectiveness under 100% adherence,^30^^,^^31^ other conditions maintain the status quo.
• Scenario 6 (Biomedical combination intervention): Increase both the overall testing rate and the treatment rate to over 50%, introduce PrEP to both LRMSM and HRMSM with coverage rates over 10%, other conditions maintain the status quo.

#### Comprehensive behavioral and biomedical interventions

• Scenario 7 (Weak comprehensive intervention): Reduce 10% of the sexual partners in the HRMSM group, increase the overall condom use rate to 40%, with 50% overall testing and treatment rate and 10% PrEP coverage rate, other conditions maintain the status quo.
• Scenario 8 (Moderate comprehensive intervention): Reduce 20% of the sexual partners in HRMSM group, increase the overall condom use rate to 50%, with 70% overall testing and treatment rate and 20% PrEP coverage rate, other conditions maintain the status quo.
• Scenario 9 (Strong comprehensive intervention): Reduce 30% of the sexual partners in HRMSM group, increase the overall condom use rate to 60%, with 90% overall testing and treatment rate and 30% PrEP coverage rate, other conditions maintain the status quo.

### Elimination analysis

We used the effective reproduction number in 2022 (*R_2022_*) as a necessary precondition for elimination, with *R_2022_*<1 indicating that transmission will begin to decline, and incidence rate<1/1000 person-years as the threshold for eliminating HIV transmission based on Granich's previous work.^32^ We used the next generation method to calculate *R_2022_* under the different scenarios. The detailed calculation process of *R_2022_* is given in Supplementary Section 2, and the calculation process for other epidemiological outcomes (such as prevalence and incidence) is given in Supplementary Section 3.

### Model calibration

Model calibration was conducted to select the most reliable model by sampling the key parameters within their possible range. The key parameters include: the percentage of MSM in the Japanese male population, number of partners, condom use rate, testing and treatment rate. Details of model calibration is given in Supplementary Section 4. All models were run using MATLAB R2019b.

### Role of the funding source

The funding source had no role in the preparation of this paper and did not have any influence on the decision to publish.

## Results

### HIV epidemic forecast under status quo

**Figure 2:**
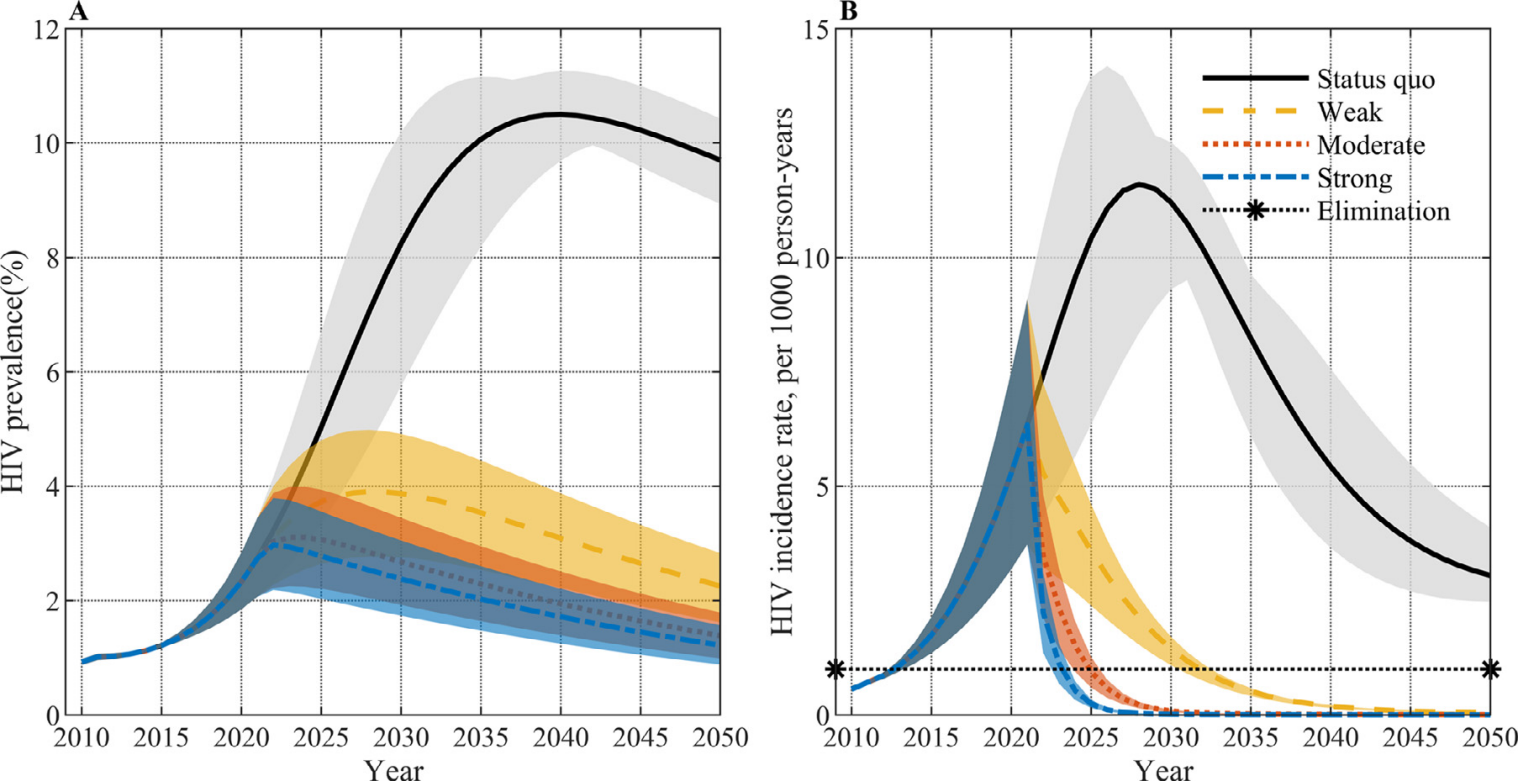
HIV prevalence (A) and HIV incidence rate (B) under status quo of current policies and three kinds of comprehensive behavior and biomedical interventions.

Figure 2 shows the trend in HIV prevalence and incidence under the current policies (solid lines). The incidence among Japanese MSM has been increasing since 2010 and will peak at 11.60 per 1000 person-years in 2028 and decline afterwards in the model. The incidence will still be greater than the threshold for eliminating HIV transmission of 1 per 1000 person-years in 2050. The prevalence has a similar trend to incidence, increasing since 2010 and peaking at 10.50% in 2040, followed by a gradual decrease. Under the status quo, *R_2022_* is expected to be 1.45 (sensitivity range 1.34 to 1.54), indicating the HIV epidemic will persist for a long time if policies are unchanged, which is consistent with the model projection.

### Effect of behavioral interventions

**Figure 3:**
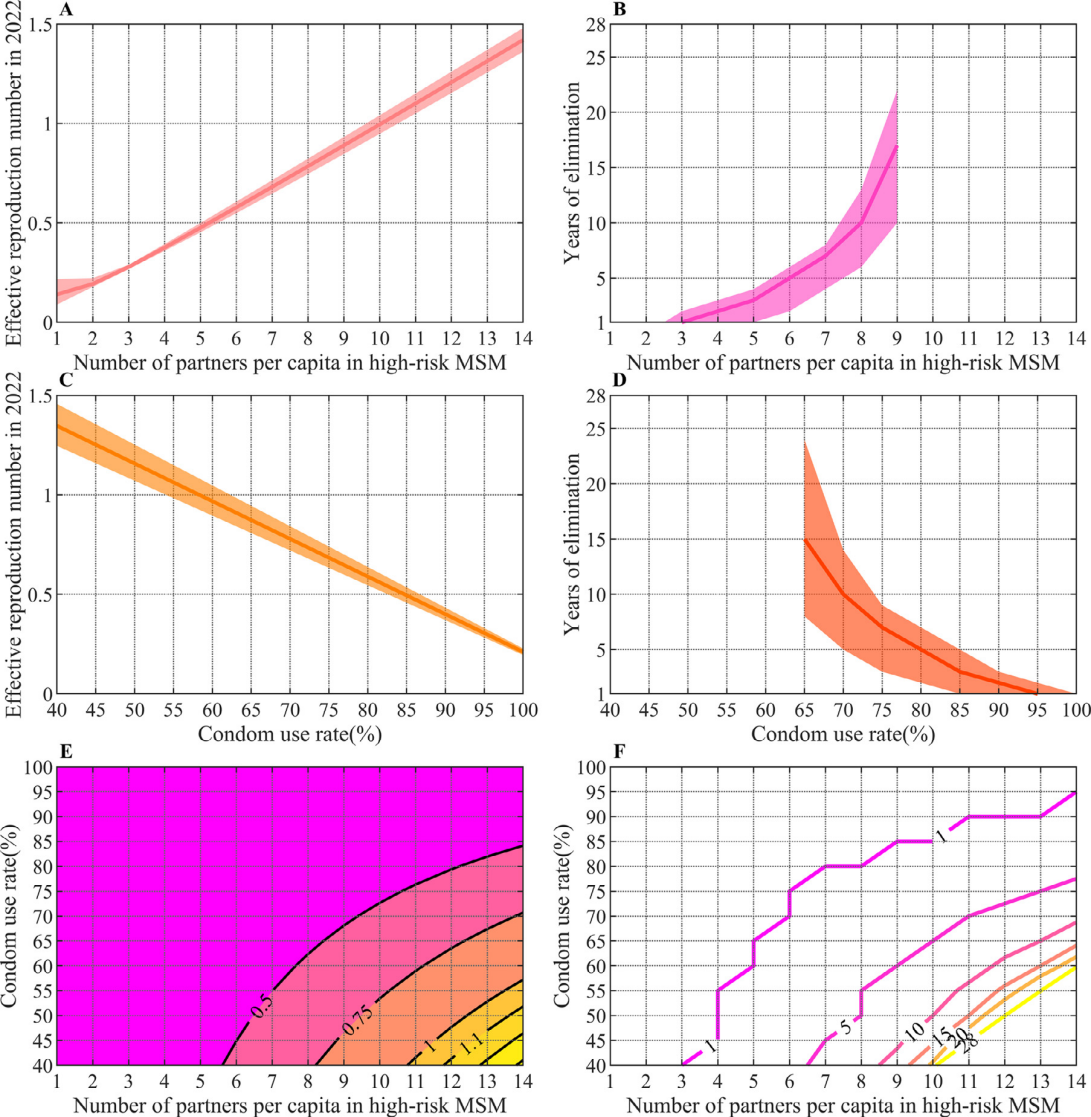
*R_2022_* (A) and the time required to eliminate HIV (B) under partner reduction intervention. **(The blank for values above 9 in the right panel indicates failure to achieve HIV elimination within 28 years (i.e., by 2050)).***R_2022_* (C) and the time required to eliminate HIV (D) under increased condom use intervention. **(The blank for values below 65% in the right panel indicates failure to achieve HIV elimination within 28 years).***R_2022_* (E) and the time required to eliminate HIV (F) under behavioral combination intervention. **(The numbers on the curves represent the value of *R_2022_* in the left panel and the time required to eliminate HIV in the right panel at the partners numbers and condom use rate corresponding to any points on the curves).**

We explored the effect of the implementation of two behavioral interventions from 2022. Figure 3A and 3B show the trend in *R_2022_* and time required to eliminate HIV transmission under different numbers of sexual partners in the HRMSM group. The value of *R_2022_* decreases with reduction in the number of sexual partners, and the time required to eliminate HIV transmission is shortened as *R_2022_* declines. When the number of sexual partners in HRMSM is reduced to less than 10, *R_2022_* will be less than 1 and transmission will be eliminated by 2050 when the annual number of sexual partners in HRMSM can be controlled under 9.

Figure 3C and 3D show the trend in *R_2022_* and time required to eliminate HIV transmission under different condom use rates. The condom use rate has a linear relationship with *R_2022_*. If overall condom use rates increase from 40% to 100%, *R_2022_* will decrease from 1.35 to less than 0.21. HIV transmission cannot be eliminated by 2050 when the condom use rate is less than 65%, but rapid elimination can be achieved when the condom use rate is higher than 80%.

Figure 3E and 3F show the trend in *R_2022_* and time required to eliminate HIV transmission under behavioral combination interventions. The numbers on the curves represent the value of *R_2022_* in the left panel and the time required to eliminate HIV in the right panel at the partners numbers and condom use rate corresponding to any points on the curves. With relatively smaller behavioral changes than single measure behavioral interventions, such as just a two partners reduction in HRMSM and 50% condom use rate, *R_2022_* can be reduced below 1 and achieve elimination by 2050.

### Effect of biomedical interventions

**Figure 4:**
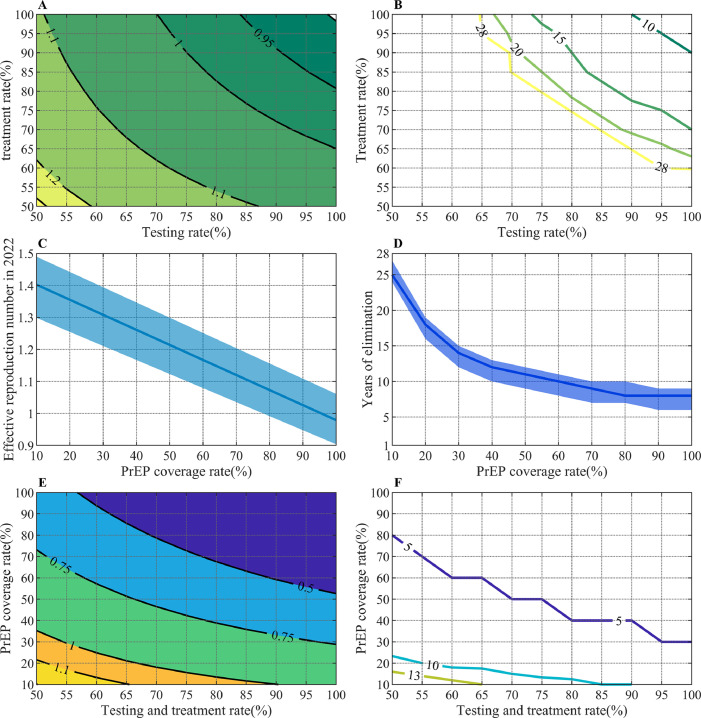
***R_2022_* (A) and the time required to eliminate HIV (B) under enhanced testing and treatment intervention.** (The numbers on the curves represent the value of *R_2022_* in the left panel and the time required to eliminate HIV in the right panel at the testing rate and treatment rate corresponding to any point on the curve). ***R_2022_* (C) and the time required to eliminate HIV (D) under introducing PrEP. *R_2022_* (E) and the time required to eliminate HIV (F) under biomedical combination intervention.** (The numbers on the curves represent the value of *R_2022_* in the left panel and the time required to eliminate HIV in the right panel at the testing and treatment rate and PrEP coverage rate corresponding to any points on the curves).

We explored the effect of introducing two different biomedical interventions from 2022. Enhanced test-and-treat strategy is an effective method to control the HIV epidemic since Japan still has low testing rates and barriers to immediate ART initiation. Figure 4A and 4B shows the trend in *R_2022_* and time required to eliminate HIV transmission under different testing rates. The range of *R_2022_* is from 1.25 to 0.90 coresponding to the annual testing rate and monthly treatment rate from 50% to 100%. *R_2022_* will be less than 1 if both testing and treatment rate are over approximately 80%. However, this will still require a long time to achieve elimination of HIV transmission, and much higher testing and treatment rates are needed to shorten the elimination time. For example, testing and treatment rates over 95% can achieve elimination of HIV transmission within 10 years.

Figure 4C and 4D show the trend in *R_2022_* and time required to eliminate HIV transmission under different PrEP coverages. PrEP coverage also has a linear relationship with *R_2022_*. As PrEP coverage increases from 10% to 100%, *R_2022_* will decrease from 1.40 to 0.97. HIV transmission will be eliminated after 25 years when there is 10% PrEP coverage, and the time required for elimination is decreased to 8 years when there is 80% coverage. Further increases in PrEP coverage beyond 80% have little additional benefit. We further analyzed the impact of PrEP for each group, finding the policy was more efficient in the high-risk group than the low-risk group, with less person-years of intervention required to avert one infection when PrEP was supplied to only the high-risk group. Detailed results are given in Supplementary Table S3.

The results of biomedical combination interventions also demonstrated the possibility that smaller changes in each in dimension could reduce *R_2022_* and achieve elimination (Figure 4E and 4F). With a limited coverage rate of PrEP, such as 10%, HIV transmission can be eliminated after 13 years with 65% testing and treatment rate, an achievement which is impossible by 2050 with a single enhanced test and treatment intervention at 65% testing and treatment rate.

### Effect of comprehensive interventions

Behavioral and biomedical interventions can achieve elimination of HIV transmission by 2050, with different intervention intensities requiring different time to achieve elimination. We explored the effect of combining both behavioral and biomedical interventions at three possible intensities. The prevalence and incidence rate under three intensities of combined intervention are shown in Figure 2. All three interventions can drastically reduce the incidence rate that is expected to continue to rise under the current policies, thereby rapidly reducing the prevalence. Even the weak combination intervention, with only 10% changes for each dimension, can play a great role in the control of the HIV epidemic. The years of elimination required under different intervention scenarios are shown in Table 2. The time to elimination required in comprehensive interventions is much less than that in each single intervention, with elimination of HIV transmission estimated to be achievable in 2032 (sensitivity range 2031 to 2033), 2025 (sensitivity range 2024 to 2026) and 2024 (sensitivity range 2023 to 2024) under weak, moderate and strong comprehensive interventions, respectively. Under the three intensities in Table 2, the time to elimination required under biomedical combination interventions (Scenario 3) is much smaller than that of behavioral combination interventions (Scenario 6), and the time gap between biomedical combination interventions and comprehensive interventions is relatively smaller, indicating that enhanced testing and treatment and PrEP play a greater role in comprehensive intervention than behavioral interventions. We also explored the time to elimination under a smaller threshold (incidence rate <1/10000 person-years), finding all three comprehensive interventions can achieve elimination of HIV transmission by 2050 but need 12 years, 5 years and 3 years longer respectively. The detailed results are presented in Supplementary Table S4.

**Table 2:** Years of elimination of HIV transmission under different intervention scenarios.

Intensity	Intervention	Year of elimination of HIV transmission						
		Scenario 1 (Sensitivity range)	Scenario 2 (Sensitivity range)	Scenario 3 (Sensitivity range)	Scenario 4 (Sensitivity range)	Scenario 5 (Sensitivity range)	Scenario 6 (Sensitivity range)	Comprehensive interventions (Sensitivity range)
Weak	Partner reduction: 10%	After 2050		After 2050				2032 (2031 – 2033)
	Condom use rate: 40%		After 2050					
	Testing and treatment: 50%				After 2050		2038 (2036 -2039)	
	PrEP coverage rate: 10%					2047 (2046 –2049)		
Moderate	Partner reduction: 20%	After 2050		2037 (2031-2043)				2025 (2024 – 2026)
	Condom use rate: 50%		After 2050					
	Testing and treatment: 70%				After 2050		2030 (2029 – 2031)	
	PrEP coverage rate: 20%					2040 (2038 – 2041)		
Strong	Partner reduction:30%	After 2050		2030 (2026 -2032)				2024 (2023 – 2024)
	Condom use rate: 60%		After 2050					
	Testing and treatment: 90%				2034 (2030 – 2037)		2027 (2026 – 2028)	
	PrEP coverage rate: 30%					2036 (2034 –2037)		

Table 3 shows the epidemiological impact under three levels of comprehensive interventions. Comprehensive interventions can prevent 81.23% to 97.48% of new HIV infections from 2022 to 2050. We also calculated the number of tests, treatments, and people taking PrEP needed in each comprehensive intervention to provide references for intervention preparation.

**Table 3:** HIV epidemic forecast under different intervention scenarios.

	Status quo	Weak intervention	Moderate intervention	Strong intervention
Epidemiological impact				
Prevalence in 2050 (%)	9.70 (8.93, 10.43)	2.25 (1.62, 2.83)	1.38 (0.99, 1.79)	1.22 (0.88, 1.57)
Incidence rate in 2050 (/1000 person-year)	3.00 (2.50, 4.10)	0.046 (0.033, 0.058)	0.0053 (0.0033, 0.0072)	0.0020 (0.0013, 0.0027)
Total HIV infections from 2022 to 2050 (× 10,000)	16.17 (11.22, 20.78)	3.04 (1.97, 4.05)	0.91 (0.53, 1.30)	0.41 (0.24, 0.60)
HIV infections prevented from 2022 to 2050 (× 10,000)	-	13.13 (9.08, 16.87)	15.26 (10.67, 19.49)	15.76 (10.98, 20.19)
HIV infections prevented from 2022 to 2050 (%)	-	81.23 (75.45, 90.17)	94.41 (92.22, 97.53)	97.48 (96.45, 98.92)
Required number of tests/treatments/people taking PrEP in the first year of the intervention (i.e., 2022)				
Number of tests required (× 100,000)	-	3.22 (3.03, 3.44)	4.17 (3.92, 4.46)	4.96 (4.67, 5.30)
Number of treatments required (× 1000)	-	3.16 (1.76, 4.63)	4.47 (2.48, 6.58)	5.68 (3.15, 8.40)
Number of PrEP required (× 10,000)	0	2.57 (2.44, 2.73)	5.79 (5.50, 6.13)	9.48 (8.99, 10.02)

## Discussion

In this study we projected the future HIV epidemic among Japanese MSM under the different intervention scenarios using a deterministic compartmental mathematical model that reflects HIV disease progression. Under current policies, our modeling found that the HIV epidemic cannot be rapidly controlled, and HIV transmission will not be eliminated by 2050, which is consistent with previous research findings.^22^^,^^33^ Although the number of new HIV notifications reported by MHLW has been declining since it peaked in 2013,^14^ the trend is based on the data of the entire population rather than the MSM population. Moreover, the decreasing number of annual newly notified cases may not reflect improvements in HIV control, but may just be the result of the recent stagnation in HIV testing.^34^

The large number of sexual partners and low condom use rates are great behavioral risks to HIV infection. This study found that to achieve HIV elimination by 2050 only with improvements in risky behaviors, the annual number of sexual partners in HRMSM needs to be reduced by at least 35% to less than 9, or condom use rates almost doubled to 65%. Strategies encouraging reduced sexual partners are controversial and past abstinence campaigns in other countries were ineffective in reducing HIV transmission at population-level due to failure to reduce partner numbers.^35^

The large reduction in partner numbers required to control HIV identified in this study shows why such programs are unlikely to be effective, and is unlikely to be a realistic goal in the Japanese context. Given the magnitude of the required partner reduction identified in this study, increasing condom use is a more realistic intervention. However, this increased condom use rate is also challenging and will require redoubled efforts. Behavioral change requires long-term efforts and public campaigns should be conducted with commitments of various kinds of stakeholders. Further enhancement of ongoing HIV education programs through multiple media^36^^,^^37^ as well as community-based approaches is needed to achieve the higher condom use rate.

Against this backdrop of increasing incidence in the status quo scenario, increasing testing and treatment rates can reduce HIV transmission risk by accelerating the process from HIV infection to receiving ART (99% of the PLWH receiving ART in Japan have achieved viral suppression.^38^). Under the current policies, it is estimated to take approximately three years (based on current testing rate) for newly-infected persons to be identified, and approximately two to five months (based on current treatment rate) for identified persons to receive treatment.^22^ The process can be shortened to approximately one year if more than 95% take annual testing and treatment initiation occurs within one month, greatly reducing the risk of infected persons being exposed to the population to spread disease. HIV prevention in Japan should incorporate more measures such as self- or home- testing^39^ to increase the testing rate and ensure immediate treatment options. Some countries have successfully shortened the time from HIV diagnosis to ART initiation,^40^ but achieving this level of testing is likely impossible. Even countries with best practice in preventing the HIV epidemic have struggled to achieve such high testing rates, with Australia achieving annual testing rates of 70.8% in gay and bisexual men in 2018^41^ and the UK achieving a rate of 67% in 2013.^42^ Models of PrEP delivery like London's Dean Street clinic offer new ways of increasing testing coverage as part of comprehensive prevention programs,^43^ but data on testing rates in their catchment population is not yet available, and Japan is yet to establish dedicated, free, anonymous clinics for sexual minorities, and until Japan adopts a model similar to those used in Australia and the UK, achieving these high rates of testing will remain a challenge.

PrEP is highly effective to prevent HIV transmission and 10% coverage with 100% adherence will enable Japan to achieve HIV elimination by 2050. Since using antiretroviral drugs for PrEP has not been officially licensed in Japan, the coverage of PrEP in the Japanese MSM population is much lower^15^ than the UK^44^ and the USA.^45^ With strong willingness to use PrEP among Japanese MSM,^15^ the government should consider approval of PrEP at reasonable prices with enough medical support and care. In particular, adherence support is essential to ensure the effectiveness of PrEP interventions.^23^ Considering that the intervention effect of PrEP is more effective in high-risk groups, in the allocation of limited PrEP resources, high-risk groups should be prioritized to improve the effectiveness of the intervention.

The study found relatively small changes in comprehensive interventions could rapidly eliminate HIV transmission. Although the change in partner numbers identified as necessary to achieve elimination was very large, a weak intervention with approximately only 10% changes in both behavioral and biomedical interventions will prevent 81.2% of new infections and achieve elimination of HIV transmission in 2032. Both behavioral and biomedical interventions are effective and could achieve HIV elimination by 2050, but isolated interventions requiring larger changes, such as those required for behavioral interventions alone, may encounter large implementation challenges in reality. Comprehensive interventions allowing small simultaneous changes could solve this problem, which not only reduce the difficulty of implementing each intervention, but also accelerate the realization of HIV elimination. Although this study cannot determine which comprehensive intervention is the most cost-effective or cost beneficial, it did find decreased additional prevention benefit in higher intensity interventions, indicating that it may not be necessary to implement strong interventions to achieve elimination. Detailed cost-effectiveness and cost-benefit studies are needed to identify the best trade-off between time and financial burden and explore the resource requirements of these interventions in the future.

Our study enables decisions to be made about the prioritization of interventions. We found that a combination of higher testing rates and more rapid entry into treatment were effective strategies, either alone or in combination, as were PrEP strategies. These combination strategies are easily achievable in theory with changes to treatment guidelines and administrative procedures for admission to treatment, but in practice they are also impeded by stigma, discrimination and exclusion that still affect sexual minorities in Japan. Continued improvements in the rights of these sexual minorities, along with reduction in stigma and marginalization, are essential to enable the most effective interventions for HIV prevention in Japan, and further efforts are needed across society to reduce the exclusion of sexual minorities.

### Limitations

The present study has several limitations. First, heterosexual transmission was not considered in the model, although a small portion of MSM have male and female partners.^28^ Since the majority (90%) of HIV transmission are among men,^14^ the lack of heterosexual transmission will not have much influence on the results. Second, the quality of parameters in the model greatly affects the outcomes. We used model calibration to select the most reliable model by sampling the key parameters within their possible ranges. However, multiple parameters were sampled simultaneously, which causes a relatively wide range of the uncertainty. Third, we used the same condom use rate, testing and treatment rate for HRMSM and LRMSM, and more detailed field investigations are needed to obtain group-specific data for these parameters that can be used to better model intervention impacts. Fourth, our study does not take into account the possibility of risk compensation among Japanese MSM, because in the absence of any current PrEP programs we are unable to determine what level of risk compensation might occur. However, in a previous study we modeled risk compensation as part of a PrEP program and found that with high adherence,^23^ even extreme levels of risk compensation (up to and including 100% risk compensation, i.e. not using any condoms at all) have no significant effect on program effectiveness. It is therefore likely that risk compensation will not be an issue in a Japanese PrEP program, provided that adherence is good. This will require that early roll-out of PrEP in Japan be accompanied by development of strategies to encourage adherence which work in the Japanese context, and there is a risk that PrEP will be less effective if such strategies are not incorporated into PrEP rollout. Finally, this study does not include the time needed to scale up the implementation of corresponding interventions, and in reality the time to achieve elimination may be longer than that predicted in our research.

## Conclusions

HIV transmission will not be eliminated by 2050 in the Japanese MSM population under current policies, and enhanced interventions are necessary to control the epidemic. Both behavioral and biomedical interventions can achieve elimination of HIV transmission by 2050, but comprehensive interventions can accelerate HIV control with high feasibility. By a small reduction in behavioral risk in Japan's most high-risk MSM populations, combined with improved testing infrastructure, improved treatment guidelines, and the introduction of PrEP, Japan can make the elimination of HIV transmission a reality within just one decade.

## Declaration of interests

All authors: No reported conflicts of interest.

## Appendix Supplementary materials

Image, application 1
