# Supplementary material for: Elimination of HIV transmission in Japanese MSM with combination interventions
Source: Lancet Reg Health West Pac. 2022 May 10;23:100467. doi: 10.1016/j.lanwpc.2022.100467 (PMC9118161; doi:10.1016/j.lanwpc.2022.100467)
Supplement: Supplementary file 1 [file mmc1.docx]

**Supplementary Material**

**Elimination of HIV transmission in Japanese MSM with combination interventions**

# Model equations

In this section, we will show the 15 ordinary differential equations and two key components in these equations: mixing parameter and transmission force.

## Ordinary differential equations

For each risk group, there are 15 ODEs describing the rate of change in the number of people in each compartment, shown in detail below. $X_{i,j}$ is the value for the population of risk group $i$ in compartment $j$. Table S1 shows the meaning of the parameters in the ODEs.

|  |
| --- |
|  |
|  |
| $\frac{dX_{i,4}}{dt}=\left(\sum_{j\geq4} \lambda_{1,j}^{i}(t)\right)X_{i,1}+\left(\sum_{j\geq4} \lambda_{2,j}^{i}(t)\right)X_{i,2}-(\psi_{4}^{i}+\theta_{4}^{i}+\mu_{4}^{i}+b_{4}^{i})X_{i,4}$ |
| $\frac{dX_{i,5}}{dt}=\left(\sum_{j\geq4} \lambda_{3,j}^{i}(t)\right)X_{i,3}+\psi_{4}^{i}X_{i,4}-(\theta_{5}^{i}+\alpha_{5}^{i}+\mu_{5}^{i}+b_{5}^{i})X_{i,5}$ |
| $\frac{dX_{i,6}}{dt}=\alpha_{5}^{i}X_{i,5}-(\theta_{6}^{i}+\mu_{6}^{i}+b_{6}^{i})X_{i,6}$ |
| $\frac{dX_{i,7}}{dt}=\theta_{4}^{i}X_{i,4}-(\psi_{7}^{i}+\theta_{7}^{i}+\mu_{7}^{i}+b_{7}^{i})X_{i,7}$ |
| $\frac{dX_{i,8}}{dt}=\theta_{5}^{i}X_{i,5}+\psi_{7}^{i}X_{i,7}-(\theta_{8}^{i}+\alpha_{8}^{i}+\mu_{8}^{i}+b_{8}^{i})X_{i,8}$ |
| $\frac{dX_{i,9}}{dt}=\theta_{6}^{i}X_{i,6}+\alpha_{8}^{i}X_{i,8}-(\theta_{9}^{i}+\mu_{9}^{i}+b_{9}^{i})X_{i,9}$ |
| $\frac{dX_{i,10}}{dt}=\theta_{7}^{i}X_{i,7}-(\psi_{10}^{i}+\theta_{10}^{i}+\mu_{10}^{i}+b_{10}^{i})X_{i,10}$ |
| $\frac{dX_{i,11}}{dt}=\theta_{8}^{i}X_{i,8}+\psi_{10}^{i}X_{i,10}-(\theta_{11}^{i}+\alpha_{11}^{i}+\mu_{11}^{i}+b_{11}^{i})X_{i,11}$ |
| $\frac{dX_{i,12}}{dt}=\theta_{9}^{i}X_{i,9}+\alpha_{11}^{i}X_{i,11}-(\theta_{12}^{i}+\mu_{12}^{i}+b_{12}^{i})X_{i,12}$ |
| $\frac{dX_{i,13}}{dt}=\theta_{10}^{i}X_{i,10}-(\psi_{13}^{i}+\mu_{13}^{i}+b_{13}^{i})X_{i,13}$ |
| $\frac{dX_{i,14}}{dt}=\theta_{11}^{i}X_{i,11}+\psi_{13}^{i}X_{i,13}-(\alpha_{14}^{i}+\mu_{14}^{i}+b_{14}^{i})X_{i,14}$ |
| $\frac{dX_{i,15}}{dt}=\theta_{12}^{i}X_{i,12}+\alpha_{14}^{i}X_{i,14}-(\mu_{15}^{i}+b_{15}^{i})X_{i,15}$ |

Table S1 Model parameters

| **Notation** | **Parameter** |
| --- | --- |
| ***Demographic*** |  |
|  | Background maturation and entry rates |
| $b_{j}^{i}$ | Annual maturation rate, male |
| $\rho_{1}^{i}$ | Annual entry rate, male |
| $\mu_{j}^{i}$ | Background mortality rate |
|  | Annual mortality rate, male |
|  | Annual mortality rate without ART |
|  | Acute |
|  | Asymptomatic (CD>500) |
|  | Asymptomatic (200<=CD4<=500) |
|  | AIDS (CD4<200) |
|  | Annual mortality rate with ART |
|  | Acute |
|  | Asymptomatic (CD>500) |
|  | Asymptomatic (200<=CD4<=500) |
|  | AIDS (CD4<200) |
| ***Biological*** |  |
|  | Duration of HIV progression status converted to months |
| $1/\theta_{j}^{i}$ | Acute to CD4>500 |
|  | CD4>500 to 200<=CD4<=500 |
|  | 200<=CD4<=500 to CD4<200 |
|  | Probability of HIV transmission per partnership, where z= acute, asymptomatic HIV and AIDS |
| $\sigma^{Z}$ | Acute (within 3 months) |
|  | Asymptomatic (CD>500) |
|  | Asymptomatic (200<=CD4<=500) |
|  | AIDS (CD4<200) |
| $r_{2}$ | Reduction in infectivity (multiplicative) due to ART |
| ***Behavioral*** |  |
|  | Annual number of partners |
| $n_{1}$ | Low risk MSM |
| $n_{2}$ | High risk MSM |
|  | Condom use (% of sexual encounters) |
| $u_{i,j}$ | Condom use rate (%) |
| $\kappa$ | Condom effectiveness |
|  | Others |
| $\varepsilon$ | Proportion of members of one group having sexual interaction with members of the other group |
| $r_{1}$ | Reduction in sexual behavior after HIV diagnosis |
| $r_{1}^{'}$ | Reduction in sexual behavior among AIDS patients |
| ***Biomedical*** |  |
|  | HIV testing |
| $\psi_{j}^{i}$ | Proportion of population tested in past 12 months, % |
|  | Rate of detection of HIV through passive case-finding |
|  | Rate of detection of AIDS through passive case-finding |
| $1/\omega_{2}^{i}$ | Average duration that uninfected individuals remain identified after testing in risk |
|  | Monthly entry rate to ART |
| $\alpha_{j}^{i}$ | Acute |
|  | Asymptomatic (CD>500) |
|  | Asymptomatic (200<=CD4<=500) |
|  | AIDS (CD4<200) |
|  | Pre-exposure prophylaxis |
|  | Rate of uninfected people start taking PrEP |
|  | Rate of PrEP dropout |
| $r_{3}$ | PrEP Effectiveness |
| ***Transmission force*** |  |
| $\sum_{j\geq4} \lambda_{k,j}^{i}(t)$ | Transmission force (k=1,2 means not in PrEP, k=3 means in PrEP) |

## Entry rate and maturation rate

The target population is MSM population aged 18-59 years old. The entry rate is defined as the rate at which people enter the target group, which means the rate at which people grow up to age 18 and start entering the model. The maturation rate is the rate of aging, which means the rate of 18-59 year olds who gradually grow to age 60 and beyond and thus are removed from the model. The formula for calculating entry rate is:

$$\rho_{1}^{i}=-\ln\left(1-\frac{18 years\text{ }old population}{18\text{-}59years\text{ }old population}\right)\text{+}growth rate$$

The formula for calculating maturation rate is:

$$b_{j}^{i}=-\ln\left(1-\frac{59 years\text{ }old population}{18\text{-}59years\text{ }old population}\right)$$

## Mixing between LRMSM and HRMSM

The model was applied to low-risk MSM (LRMSM) and high-risk MSM (HRMSM), respectively. People in each group have a chance to interact with people in the other group. To model the chance, a mixing parameter $\varepsilon$ was introduced, with $1-\varepsilon$ representing the fraction of partnerships occurring within one’s own group. The proportion of the partnerships that members of LRMSM have with members of LRMSM is:

$$\eta_{1,1}\text{=}(1-\varepsilon)+\varepsilon\frac{TP1}{TP1+TP2}$$

The proportion of the partnerships that members of LRMSM have with members of HRMSM is:

$$\eta_{1,2}\text{=}\varepsilon\frac{TP2}{TP1+TP2}$$

The proportion of the partnerships that members of HRMSM have with members of LRMSM is:

$$\eta_{2,1}\text{=}\varepsilon\frac{TP1}{TP1+TP2}$$

The proportion of the partnerships that members of HRMSM have with members of HRMSM is:

$$\eta_{2,2}\text{=}(1-\varepsilon)+\varepsilon\frac{TP2}{TP1+TP2}$$

Where, $TP1$and $TP2$are the total numbers of partnerships of LRMSM and HRMSM respectively.

$$TP1=\sum_{i=1} \left[(\sum_{j=1,2,3,4,7,10} X_{i,j})n_{i}+(\sum_{j=5,6,8,9,11,12} X_{i,j})n_{i}(1-r_{1})+(\sum_{j=13,14,15} X_{i,j})n_{i}(1-r_{1}')\right]$$

$$TP2=\sum_{i=2} \left[(\sum_{j=1,2,3,4,7,10} X_{i,j})n_{i}+(\sum_{j=5,6,8,9,11,12} X_{i,j})n_{i}(1-r_{1})+(\sum_{j=13,14,15} X_{i,j})n_{i}(1-r_{1}')\right]$$

## Transmission force

Transmission force represents the rate of uninfected people $\left(j=1,2,3\right)$ entering the infected population$\left(j=4,5\right)$, which is the sum of force of infections with each infectious compartment$\left(j\text{=}4,5,\cdots,15\right)$. The detailed calculations are described in a previous paper[^1^](#_ENREF_1).

Transmission forces for LRMSM that are not in PrEP are:

$$\sum_{j\geq4} \lambda_{1,j}^{1}(t)=\sum_{j\geq4} \lambda_{2,j}^{1}(t)=\sum_{j\geq4} \left\{\left.1-N_{-}LRMSM_{j}^{\eta_{1,1}n_{1}(1-u_{1}\kappa)}\right\}\right.+\sum_{j\geq4} \left\{\left.1-N_{-}HRMSM_{j}^{\eta_{1,2}n_{1}(1-u_{1}\kappa)}\right\}\right.$$

Transmission force for LRMSM that are in PrEP is:

$$\sum_{j\geq4} \lambda_{3,j}^{1}(t)=\sum_{j\geq4} \left\{\left.1-N_{-}prepLRMSM_{j}^{\eta_{1,1}n_{1}(1-u_{1}\kappa)}\right\}\right.+\sum_{j\geq4} \left\{\left.1-N_{-}prepHRMSM_{j}^{\eta_{1,2}n_{1}(1-u_{1}\kappa)}\right\}\right.$$

Transmission forces for HRMSM that are not in PrEP are:

$$\sum_{j\geq4} \lambda_{1,j}^{2}(t)=\sum_{j\geq4} \lambda_{2,j}^{2}(t)=\sum_{j\geq4} \left\{\left.1-N_{-}LRMSM_{j}^{\eta_{2,1}n_{2}(1-u_{2}\kappa)}\right\}\right.+\sum_{j\geq4} \left\{\left.1-N_{-}HRMSM_{j}^{\eta_{2,2}n_{2}(1-u_{2}\kappa)}\right\}\right.$$

Transmission force for HRMSM that are in PrEP is:

$$\sum_{j\geq4} \lambda_{3,j}^{2}(t)=\sum_{j\geq4} \left\{\left.1-N_{-}prepLRMSM_{j}^{\eta_{2,1}n_{2}(1-u_{2}\kappa)}\right\}\right.+\sum_{j\geq4} \left\{\left.1-N_{-}prepHRMSM_{j}^{\eta_{2,2}n_{2}(1-u_{2}\kappa)}\right\}\right.$$

Where$N_{-}LRMSM_{j}\left(j\text{=}4,5,\cdots,15\right)$is the probability that MSM without PrEP are not infected by LRMSM in compartment j in one partnership. $\begin{matrix} N_{-}LRMSM_{4}=\left[1-\left(\frac{X_{1,4}n_{1}}{TP1}\sigma^{acute}\right)\right];N_{-}LRMSM_{10}=\left[1-\left(\frac{X_{1,10}n_{1}}{TP1}\sigma^{asym}\right)\right]; \\ N_{-}LRMSM_{5}=\left[1-\left(\frac{X_{1,5}n_{1}(1-r_{1})}{TP1}\sigma^{acute}\right)\right];N_{-}LRMSM_{11}=\left[1-\left(\frac{X_{1,11}n_{1}(1-r_{1})}{TP1}\sigma^{asym}\right)\right]; \\ N_{-}LRMSM_{6}=\left[1-\left(\frac{X_{1,6}n_{1}(1-r_{1})}{TP1}\sigma^{acute}(1-r_{2})\right)\right];N_{-}LRMSM_{12}=\left[1-\left(\frac{X_{1,12}n_{1}(1-r_{1})}{TP1}\sigma^{asym}(1-r_{2})\right)\right]; \\ N_{-}LRMSM_{7}=\left[1-\left(\frac{X_{1,7}n_{1}}{TP1}\sigma^{asym}\right)\right];N_{-}LRMSM_{13}=\left[1-\left(\frac{X_{1,13}n_{1}(1-r_{1}^{'})}{TP1}\sigma^{AIDS}\right)\right]; \\ N_{-}LRMSM_{8}=\left[1-\left(\frac{X_{1,8}n_{1}(1-r_{1})}{TP1}\sigma^{asym}\right)\right];N_{-}LRMSM_{14}=\left[1-\left(\frac{X_{1,14}n_{1}(1-r_{1}^{'})}{TP1}\sigma^{AIDS}\right)\right]; \\ N_{-}LRMSM_{9}=\left[1-\left(\frac{X_{1,9}n_{1}(1-r_{1})}{TP1}\sigma^{asym}(1-r_{2})\right)\right];N_{-}LRMSM_{15}=\left[1-\left(\frac{X_{1,15}n_{1}(1-r_{1}^{'})}{TP1}\sigma^{AIDS}(1-r_{2})\right)\right] \end{matrix}$

$N_{-}prepLRMSM_{j}\left(j\text{=}4,5,\cdots,15\right)$ is the probability that MSM with PrEP are not infected by LRMSM in compartment j in one partnership.

$$\begin{matrix} N_{-}prepLRMSM_{4}=\left[1-\left(\frac{X_{1,4}n_{1}}{TP1}\sigma^{acute}(1-r_{3})\right)\right];N_{-}prepLRMSM_{10}=\left[1-\left(\frac{X_{1,10}n_{1}}{TP1}\sigma^{asym}(1-r_{3})\right)\right]; \\ N_{-}prepLRMSM_{5}=\left[1-\left(\frac{X_{1,5}n_{1}(1-r_{1})}{TP1}\sigma^{acute}(1-r_{3})\right)\right];N_{-}prepLRMSM_{11}=\left[1-\left(\frac{X_{1,11}n_{1}(1-r_{1})}{TP1}\sigma^{asym}(1-r_{3})\right)\right]; \\ N_{-}prepLRMSM_{6}=\left[1-\left(\frac{X_{1,6}n_{1}(1-r_{1})}{TP1}\sigma^{acute}(1-r_{2})(1-r_{3})\right)\right];N_{-}prepLRMSM_{12}=\left[1-\left(\frac{X_{1,12}n_{1}(1-r_{1})}{TP1}\sigma^{asym}(1-r_{2})(1-r_{3})\right)\right]; \\ N_{-}prepLRMSM_{7}=\left[1-\left(\frac{X_{1,7}n_{1}}{TP1}\sigma^{asym}(1-r_{3})\right)\right];N_{-}prepLRMSM_{13}=\left[1-\left(\frac{X_{1,13}n_{1}(1-r_{1}^{'})}{TP1}\sigma^{AIDS}(1-r_{3})\right)\right]; \\ N_{-}prepLRMSM_{8}=\left[1-\left(\frac{X_{1,8}n_{1}(1-r_{1})}{TP1}\sigma^{asym}(1-r_{3})\right)\right];N_{-}prepLRMSM_{14}=\left[1-\left(\frac{X_{1,14}n_{1}(1-r_{1}^{'})}{TP1}\sigma^{AIDS}(1-r_{3})\right)\right]; \\ N_{-}prepLRMSM_{9}=\left[1-\left(\frac{X_{1,9}n_{1}(1-r_{1})}{TP1}\sigma^{asym}(1-r_{2})(1-r_{3})\right)\right];N_{-}prepLRMSM_{15}=\left[1-\left(\frac{X_{1,15}n_{1}(1-r_{1}^{'})}{TP1}\sigma^{AIDS}(1-r_{2})(1-r_{3})\right)\right] \end{matrix}$$

$N_{-}HRMSM_{j}\left(j\text{=}4,5,\cdots,15\right)$ is the probability that MSM without PrEP are not infected by HRMSM in compartment j in one partnership.

$$\begin{matrix} N_{-}HRMSM_{4}=\left[1-\left(\frac{X_{2,4}n_{2}}{TP2}\sigma^{acute}\right)\right];N_{-}HRMSM_{10}=\left[1-\left(\frac{X_{2,10}n_{2}}{TP2}\sigma^{asym}\right)\right]; \\ N_{-}HRMSM_{5}=\left[1-\left(\frac{X_{2,5}n_{2}(1-r_{1})}{TP2}\sigma^{acute}\right)\right];N_{-}HRMSM_{11}=\left[1-\left(\frac{X_{2,11}n_{2}(1-r_{1})}{TP2}\sigma^{asym}\right)\right]; \\ N_{-}HRMSM_{6}=\left[1-\left(\frac{X_{2,6}n_{2}(1-r_{1})}{TP2}\sigma^{acute}(1-r_{2})\right)\right];N_{-}HRMSM_{12}=\left[1-\left(\frac{X_{2,12}n_{2}(1-r_{1})}{TP2}\sigma^{asym}(1-r_{2})\right)\right]; \\ N_{-}HRMSM_{7}=\left[1-\left(\frac{X_{2,7}n_{2}}{TP2}\sigma^{asym}\right)\right];N_{-}HRMSM_{13}=\left[1-\left(\frac{X_{2,13}n_{2}(1-r_{1}^{'})}{TP2}\sigma^{AIDS}\right)\right]; \\ N_{-}HRMSM_{8}=\left[1-\left(\frac{X_{2,8}n_{2}(1-r_{1})}{TP2}\sigma^{asym}\right)\right];N_{-}HRMSM_{14}=\left[1-\left(\frac{X_{2,14}n_{2}(1-r_{1}^{'})}{TP2}\sigma^{AIDS}\right)\right]; \\ N_{-}HRMSM_{9}=\left[1-\left(\frac{X_{2,9}n_{2}(1-r_{1})}{TP2}\sigma^{asym}(1-r_{2})\right)\right];N_{-}HRMSM_{15}=\left[1-\left(\frac{X_{2,15}n_{2}(1-r_{1}^{'})}{TP2}\sigma^{AIDS}(1-r_{2})\right)\right] \end{matrix}$$

$N_{-}prepHRMSM_{j}\left(j\text{=}4,5,\cdots,15\right)$is the probability that MSM with PrEP are not infected by HRMSM in compartment j in one partnership.

$$\begin{matrix} N_{-}prepHRMSM_{4}=\left[1-\left(\frac{X_{2,4}n_{2}}{TP2}\sigma^{acute}(1-r_{3})\right)\right];N_{-}prepHRMSM_{10}=\left[1-\left(\frac{X_{2,10}n_{2}}{TP2}\sigma^{asym}(1-r_{3})\right)\right]; \\ N_{-}prepHRMSM_{5}=\left[1-\left(\frac{X_{2,5}n_{2}(1-r_{1})}{TP2}\sigma^{acute}(1-r_{3})\right)\right];N_{-}prepHRMSM_{11}=\left[1-\left(\frac{X_{2,11}n_{2}(1-r_{1})}{TP2}\sigma^{asym}(1-r_{3})\right)\right]; \\ N_{-}prepHRMSM_{6}=\left[1-\left(\frac{X_{2,6}n_{2}(1-r_{1})}{TP2}\sigma^{acute}(1-r_{2})(1-r_{3})\right)\right];N_{-}prepHRMSM_{12}=\left[1-\left(\frac{X_{2,12}n_{2}(1-r_{1})}{TP2}\sigma^{asym}(1-r_{2})(1-r_{3})\right)\right]; \\ N_{-}prepHRMSM_{7}=\left[1-\left(\frac{X_{2,7}n_{2}}{TP2}\sigma^{asym}(1-r_{3})\right)\right];N_{-}prepHRMSM_{13}=\left[1-\left(\frac{X_{2,13}n_{2}(1-r_{1}^{'})}{TP2}\sigma^{AIDS}(1-r_{3})\right)\right]; \\ N_{-}prepHRMSM_{8}=\left[1-\left(\frac{X_{2,8}n_{2}(1-r_{1})}{TP2}\sigma^{asym}(1-r_{3})\right)\right];N_{-}prepHRMSM_{14}=\left[1-\left(\frac{X_{2,14}n_{2}(1-r_{1}^{'})}{TP2}\sigma^{AIDS}(1-r_{3})\right)\right]; \\ N_{-}prepHRMSM_{9}=\left[1-\left(\frac{X_{2,9}n_{2}(1-r_{1})}{TP2}\sigma^{asym}(1-r_{2})(1-r_{3})\right)\right];N_{-}prepHRMSM_{15}=\left[1-\left(\frac{X_{2,15}n_{2}(1-r_{1}^{'})}{TP2}\sigma^{AIDS}(1-r_{2})(1-r_{3})\right)\right] \end{matrix}$$

# Effective reproduction number of HIV

The reproduction number ($R$) is an important concept in the field of epidemiology and infectious diseases study. $R$ is the average number of secondary cases that one case would produce in her/his/its lifetime. When $R$>1, the epidemic will continue, while when$R$<1, the epidemic will slow down and can be expected to end in the future. The basic reproduction number $R_{0}$ is the reproduction number in a completely susceptible population[^2^](#_ENREF_2), while the effective reproduction number $R_{t}$at time $t$ is used when there is a certain amount of infections in the population or some interventions have been implemented[^3^](#_ENREF_3).

In this section, first we used the next generation matrix method to obtain the formula of $R_{0}^{i}$ for each group without considering the interaction between two groups, and then applied a meta-population approach to obtain a formula for $R_{0}$ for the whole MSM population by incorporating the mixing function. Finally, we derived the effective reproduction number $R_{t}$ by applying the formula of $R_{0}$ but using the parameter values in year $t$.

## Next generation matrix method

The next generation matrix method was applied to the deterministic compartment model for each group[^4^](#_ENREF_4). In this method each of the ODE of each infected compartment is divided into two processes: new infections in group $i$ and infected compartment $j$, $f_{j}^{i}$ and transition in group $i$ in infected compartments $j$,$v_{j}^{i}$.

$$\frac{dX_{i,j}}{dt}=f_{j}^{i}(X_{0}^{i})-v_{j}^{i}(X_{0}^{i}),j\text{=}4,5,\cdots,15$$

Where$X_{0}^{i}$is the disease-free equilibrium (DFE), which assumes there are no infected cases in the population, and the number of people in the uninfected group is equal to the population. The next generation matrix for group $i$ is defined as

$$N=F^{i}(V^{i})^{-1}$$

where Jacobian matrices $F^{i}$ and $V^{i}$ are:

Where $j,k\text{=}4,5,\cdots,15$. The basic reproduction number$R_{0}^{i}$is the maximum eigenvalue of the next generation matrix in equation 33.

### Next generation matrix for LRMSM

The new infection vector$f^{1}$has 12 elements, which can be expressed as:

$$f^{1}=\left[\sum_{j\geq4} \lambda_{1,j}^{1}(t)(X_{1,1}+X_{1,2}),\sum_{j\geq4} \lambda_{3,j}^{1}(t)X_{1,3},0,0,\cdots,0\right]$$

Interactions between two groups are not considered in the calculation of a single group. Therefore, the transmission force (equations 24 and 25) can be rewritten as

$$\sum_{j\geq4} \lambda_{1,j}^{1}(t)=\sum_{j\geq4} \left\{\left.1-N_{-}LRMSM_{j}^{n_{1}(1-u_{1}\kappa)}\right\}\right.$$

$$\sum_{j\geq4} \lambda_{3,j}^{1}(t)=\sum_{j\geq4} \left\{\left.1-N_{-}prepLRMSM_{j}^{^{n_{1}(1-u_{1}\kappa)}}\right\}\right.$$

According to equations 28 and 29, the simplified form of $N_{-}LRMSM_{j}$ and $N_{-}prepLRMSM_{j}$can be rewritten as $\text{1-}\frac{LR(X_{1,j})}{TP_{1}}$ and $\text{1-}\frac{prepLR(X_{1,j})}{TP_{1}}$ respectively. When the system is at DFE, $TP_{1}=(X_{1,1}+X_{1,2}+X_{1,3})n_{1}=N_{1}n_{1}$, and $N_{1}$is the number of LRMSM. In order to obtain the new infection Jacobian matrix $F^{1}$, we need to calculate the partial derivative of each element of $f^{1}$ with respect to every$X_{1,j}(j\text{=}4,5,\cdots,15)$, the key to which lies in the first two elements of $f^{1}$, because the partial derivatives of the third to twelfth elements are all 0 (see equation 36). The partial derivative of $\sum_{j\geq4} \lambda_{1,j}^{1}(t)$:

$$\begin{matrix} \frac{\partial}{\partial X_{1,k}}\sum_{j\geq4} \left\{\left.1-N_{-}LRMSM_{j}^{n_{1}(1-u_{1}\kappa)}\right\}\right. \\ =\sum_{j\geq4} \frac{\partial}{\partial X_{1,k}}\left\{\left.1-N_{-}LRMSM_{j}^{n_{1}(1-u_{1}\kappa)}\right\}\right. \\ =\sum_{j\geq4} \frac{\partial}{\partial X_{1,k}}\left\{\left.1-\left(1-\frac{LR(X_{1,j})}{TP_{1}}\right)^{n_{1}(1-u_{1}\kappa)}\right\}\right. \\ =-\sum_{j\geq4} \frac{\partial}{\partial X_{1,k}}\left(1-\frac{LR(X_{1,j})}{TP_{1}}\right)^{n_{1}(1-u_{1}\kappa)} \\ =-\sum_{j\geq4} \left(n_{1}(1-u_{1}\kappa)\left(1-\frac{LR(X_{1,j})}{TP_{1}}\right)^{n_{1}(1-u_{1}\kappa)-1}\right)\left(-\frac{\frac{\partial LR(X_{1,j})}{\partial X_{1,k}}TP_{1}-LR(X_{1,j})\frac{\partial TP_{1}}{\partial X_{1,k}}}{TP_{1}^{2}}\right) \end{matrix}$$

The partial derivative of $\sum_{j\geq4} \lambda_{3,j}^{1}(t)$:

$$\begin{matrix} \frac{\partial}{\partial X_{1,k}}\sum_{j\geq4} \left\{\left.1-N_{-}prepLRMSM_{j}^{n_{1}(1-u_{1}\kappa)}\right\}\right. \\ =\sum_{j\geq4} \frac{\partial}{\partial X_{1,k}}\left\{\left.1-N_{-}prepLRMSM_{j}^{n_{1}(1-u_{1}\kappa)}\right\}\right. \\ =\sum_{j\geq4} \frac{\partial}{\partial X_{1,k}}\left\{\left.1-\left(1-\frac{prepLR(X_{1,j})}{TP_{1}}\right)^{n_{1}(1-u_{1}\kappa)}\right\}\right. \\ =-\sum_{j\geq4} \frac{\partial}{\partial X_{1,k}}\left(1-\frac{prepLR(X_{1,j})}{TP_{1}}\right)^{n_{1}(1-u_{1}\kappa)} \\ =-\sum_{j\geq4} \left(n_{1}(1-u_{1}\kappa)\left(1-\frac{prepLR(X_{1,j})}{TP_{1}}\right)^{n_{1}(1-u_{1}\kappa)-1}\right)\left(-\frac{\frac{\partial prepLR(X_{1,j})}{\partial X_{1,k}}TP_{1}-prepLR(X_{1,j})\frac{\partial TP_{1}}{\partial X_{1,k}}}{TP_{1}^{2}}\right) \end{matrix}$$

Because both $LR(X_{1,j})$ and $prepLR(X_{1,j})$ only depend on the number of people in compartment $j$, $LR(X_{1,j})\propto X_{1,j}$and $prepLR(X_{1,j})\propto X_{1,j}$, therefore,

Applying equations 41 and 42 to equations 39 and 40, we can simplify equations 39 and 40 to

According to equations 28 and 29, $\frac{\partial LR(X_{1,k})}{\partial X_{1,k}}$ and $\frac{\partial prepLR(X_{1,k})}{\partial X_{1,k}}$are easy to calculate.

According to the calculation process above, define the Jacobian matrix $F^{1}$as:

$$F_{j-3,k-3}^{1}=\left[\frac{\partial f_{j-3}^{1}}{\partial X_{1,k}}\right]=\left\{\begin{matrix} \beta_{j-3,k-3}^{1},if\text{ 4}\leq j\leq5 \\ 0,\text{ }if\text{ 5<}j\leq15 \end{matrix}\right.\text{，}k\in(4,15)$$

Where $\beta^{1}$ is:

The transition vector $v^{1}$ consists of the differentiation part of each infected compartments (equation 4 to equation 15), except for the new infection parts. Because annual maturation rate $b_{j}^{i}$, annual mortality rate $\mu_{j}^{i}$, HIV progression rate $\theta_{j}^{i}$, HIV testing rate $\psi_{j}^{i}$ are the same in both risk groups, we omit the superscript in the formula. The equation for transition vector $v^{1}$ is shown in equation 48.

$$\nu^{1}\text{=}\left[\begin{matrix} \left(\psi_{4}+\theta_{4}+\mu_{4}+b_{4}\right)X_{1,4} \\ -\psi_{4}X_{1,4}+\left(\theta_{5}+\alpha_{5}+\mu_{5}+b_{5}\right)X_{1,5} \\ -\alpha_{5}X_{1,5}+(\theta_{6}+\mu_{6}+b_{6})X_{1,6} \\ -\theta_{4}X_{1,4}+(\psi_{7}+\theta_{7}+\mu_{7}+b_{7})X_{1,7} \\ -\theta_{5}X_{1,5}-\psi_{7}X_{1,7}+(\theta_{8}+\alpha_{8}+\mu_{8}+b_{8})X_{1,8} \\ -\theta_{6}X_{1,6}-\alpha_{8}X_{1,8}+(\theta_{9}+\mu_{9}+b_{9})X_{1,9} \\ -\theta_{7}X_{1,7}+(\psi_{10}+\theta_{10}+\mu_{10}+b_{10})X_{1,10} \\ -\theta_{8}X_{1,8}-\psi_{10}X_{1,10}+(\theta_{11}+\alpha_{11}+\mu_{11}+b_{11})X_{1,11} \\ -\theta_{9}X_{1,9}-\alpha_{11}X_{1,11}+(\theta_{12}+\mu_{12}+b_{12})X_{1,12} \\ -\theta_{10}X_{1,10}+(\psi_{13}+\mu_{13}+b_{13})X_{1,13} \\ -\theta_{11}X_{1,11}-\psi_{13}X_{1,13}+(\alpha_{14}+\mu_{14}+b_{14})X_{1,14} \\ -\theta_{12}X_{1,12}-\alpha_{14}X_{1,14}+(\mu_{15}+b_{15})X_{1,15} \end{matrix}\right]$$

The transition Jacobian matrixs $V^{1}$is derived from this vector, as the matrix of partial derivatives:

$$V^{1}=\left[\begin{matrix} \psi_{4}+\theta_{4}+\mu_{4}+b_{4} & 0 & 0 & 0 & 0 & 0 & 0 & 0 & 0 & 0 & 0 & 0 \\ -\psi_{4} & \theta_{5}+\alpha_{5}+\mu_{5}+b_{5} & 0 & 0 & 0 & 0 & 0 & 0 & 0 & 0 & 0 & 0 \\ 0 & -\alpha_{5} & \theta_{6}+\mu_{6}+b_{6} & 0 & 0 & 0 & 0 & 0 & 0 & 0 & 0 & 0 \\ -\theta_{4} & 0 & 0 & \psi_{7}+\theta_{7}+\mu_{7}+b_{7} & 0 & 0 & 0 & 0 & 0 & 0 & 0 & 0 \\ 0 & -\theta_{5} & 0 & -\psi_{7} & \theta_{8}+\alpha_{8}+\mu_{8}+b_{8} & 0 & 0 & 0 & 0 & 0 & 0 & 0 \\ 0 & 0 & -\theta_{6} & 0 & -\alpha_{8} & \theta_{9}+\mu_{9}+b_{9} & 0 & 0 & 0 & 0 & 0 & 0 \\ 0 & 0 & 0 & -\theta_{7} & 0 & 0 & \psi_{10}+\theta_{10}+\mu_{10}+b_{10} & 0 & 0 & 0 & 0 & 0 \\ 0 & 0 & 0 & 0 & -\theta_{8} & 0 & -\psi_{10} & \theta_{11}+\alpha_{11}+\mu_{11}+b_{11} & 0 & 0 & 0 & 0 \\ 0 & 0 & 0 & 0 & 0 & -\theta_{9} & 0 & -\alpha_{11} & \theta_{12}+\mu_{12}+b_{12} & 0 & 0 & 0 \\ 0 & 0 & 0 & 0 & 0 & 0 & -\theta_{10} & 0 & 0 & \psi_{13}+\mu_{13}+b_{13} & 0 & 0 \\ 0 & 0 & 0 & 0 & 0 & 0 & 0 & -\theta_{11} & 0 & -\psi_{13} & \alpha_{14}+\mu_{14}+b_{14} & 0 \\ 0 & 0 & 0 & 0 & 0 & 0 & 0 & 0 & -\theta_{12} & 0 & -\alpha_{14} & \mu_{15}+b_{15} \end{matrix}\right]$$

The basic reproduction number for the LRMSM group, $R_{0}^{1}$, is the maximum eigenvalue of $F^{1}(V^{1})^{-1}$.

### Next generation matrix for HRMSM

The new infection vector$f^{2}$has 12 elements, which can be written as:

$$f^{2}=\left[\sum_{j\geq4} \lambda_{1,j}^{2}(t)(X_{2,1}+X_{2,2}),\sum_{j\geq4} \lambda_{3,j}^{2}(t)X_{2,3},0,0,\cdots,0\right]$$

Interactions between two groups are not considered in the calculation of a single group. Therefore, the transmission force (equations 26 and 27) can be rewritten as

According to equations 30 and 31, the simplified form of $N_{-}HRMSM_{j}$ and $N_{-}prepHRMSM_{j}$can be rewritten as $\text{1-}\frac{HR(X_{2,j})}{TP_{2}}$ and $\text{1-}\frac{prepHR(X_{2,j})}{TP_{2}}$ respectively. When the system is at DFE, $TP_{2}=(X_{2,1}+X_{2,2}+X_{2,3})n_{2}=N_{2}n_{2}$, where $N_{2}$is the number of LRMSM. In order to obtain new infection Jacobian matrix $F^{2}$, we need to calculate the partial derivative of each element of $f^{2}$with respect to every $X_{2,j}(j\text{=}4,5,\cdots,15)$, and the key to calculate the partial derivatives lies in the first two elements of $f^{2}$, because the derivatives for third to twelfth elements are 0 (see equation 50). The partial derivative of $\sum_{j\geq4} \lambda_{1,j}^{2}(t)$:

$$\begin{matrix} \frac{\partial}{\partial X_{2,k}}\sum_{j\geq4} \left\{\left.1-N_{-}HRMSM_{j}^{n_{2}(1-u_{2}\kappa)}\right\}\right. \\ =\sum_{j\geq4} \frac{\partial}{\partial X_{2,k}}\left\{\left.1-N_{-}HRMSM_{j}^{n_{2}(1-u_{2}\kappa)}\right\}\right. \\ =\sum_{j\geq4} \frac{\partial}{\partial X_{2,k}}\left\{\left.1-\left(1-\frac{HR(X_{2,j})}{TP_{2}}\right)^{n_{2}(1-u_{2}\kappa)}\right\}\right. \\ =-\sum_{j\geq4} \frac{\partial}{\partial X_{2,k}}\left(1-\frac{HR(X_{2,j})}{TP_{2}}\right)^{n_{2}(1-u_{2}\kappa)} \\ =-\sum_{j\geq4} \left(n_{2}(1-u_{2}\kappa)\left(1-\frac{HR(X_{2,j})}{TP_{2}}\right)^{n_{2}(1-u_{2}\kappa)-1}\right)\left(-\frac{\frac{\partial HR(X_{2,j})}{\partial X_{2,k}}TP_{2}-HR(X_{2,j})\frac{\partial TP_{2}}{\partial X_{2,k}}}{TP_{2}^{2}}\right) \end{matrix}$$

The partial derivative of $\sum_{j\geq4} \lambda_{3,j}^{2}(t)$:

$$\begin{matrix} \frac{\partial}{\partial X_{2,k}}\sum_{j\geq4} \left\{\left.1-N_{-}prepHRMSM_{j}^{n_{2}(1-u_{2}\kappa)}\right\}\right. \\ =\sum_{j\geq4} \frac{\partial}{\partial X_{2,k}}\left\{\left.1-N_{-}prepHRMSM_{j}^{n_{2}(1-u_{2}\kappa)}\right\}\right. \\ =\sum_{j\geq4} \frac{\partial}{\partial X_{2,k}}\left\{\left.1-\left(1-\frac{prepHR(X_{2,j})}{TP_{2}}\right)^{n_{2}(1-u_{2}\kappa)}\right\}\right. \\ =-\sum_{j\geq4} \frac{\partial}{\partial X_{2,k}}\left(1-\frac{prepHR(X_{2,j})}{TP_{2}}\right)^{n_{2}(1-u_{2}\kappa)} \\ =-\sum_{j\geq4} \left(n_{2}(1-u_{2}\kappa)\left(1-\frac{prepHR(X_{2,j})}{TP_{2}}\right)^{n_{2}(1-u_{2}\kappa)-1}\right)\left(-\frac{\frac{\partial prepHR(X_{2,j})}{\partial X_{2,k}}TP_{2}-prepHR(X_{2,j})\frac{\partial TP_{2}}{\partial X_{2,k}}}{TP_{2}^{2}}\right) \end{matrix}$$

Because both $HR(X_{2,j})$ and $prepHR(X_{2,j})$ only depend on the number of people in compartment $j$, $HR(X_{2,j})\propto X_{2,j}$and $prepHR(X_{2,j})\propto X_{2,j}$, therefore,

Applying equations 55 and 56 to equations 53 and 54, equations 53 and 54 can be simplified to

According to equations 30 and 31, $\frac{\partial HR(X_{2,k})}{\partial X_{2,k}}$and $\frac{\partial prepHR(X_{2,k})}{\partial X_{2,k}}$are easy to calculate.

According to the calculation process above, define Jacobian matrix $F^{2}$as:

$$F_{j-3,k-3}^{2}=\left[\frac{\partial f_{j-3}^{2}}{\partial X_{2,k}}\right]=\left\{\begin{matrix} \beta_{j-3,k-3}^{2},if\text{ 4}\leq j\leq5 \\ 0,\text{ }if\text{ 5<}j\leq15 \end{matrix}\right.\text{，}k\in(4,15)$$

Where $\beta^{2}$ is:

Transition vector $v^{2}$ is the same as $v^{1}$, which does not change with the group. Therefore, $V^{2}=V^{1}$, and the basic reproduction number of HRMSM $R_{0}^{2}$ is the maximum eigenvalue of $F^{2}(V^{2})^{-1}$.

## Meta-population reproduction number calculation approach

When the whole population is divided into several sub-populations, and there are interactions among these sub-populations, the meta-population reproduction number approach is needed. The approach of van den Driessche and Watmough was used to formulate the next generation matrix $K$ for the whole population in the study[^5^](#_ENREF_5), considering the mixing between LRMSM and HRMSM, based on the following formula:

$$K=\left[\begin{matrix} R_{0}^{1}\eta_{1,1} & R_{0}^{1}\eta_{1,2} \\ R_{0}^{2}\eta_{2,1} & R_{0}^{2}\eta_{2,2} \end{matrix}\right]$$

The basic reproduction number $R_{0}$ for the whole population is the maximum eigenvalue of $K$, where $\eta_{i,j}$ (equations 18 to 21) is the proportion of the partnerships that members of group $i$ have with members of group $j$.

## Effective reproduction number

According to sub-section 2.1 and 2.2, we obtained the formula of basic reproduction number $R_{0}$. The effective reproduction number $R_{t}$can be obtained by applying the formula of $R_{0}$ but using the parameter values in year $t$. Because the solution to the next generation matrix method for this mathematical model is complex, no analytic expression could be obtained, so we used numerical simulation to estimate values of $R_{2022}$ under the different scenarios.

# Model outcomes

In this section, we will show the calculation of model outcomes. The time unit of the model was months, the parameter values given by year were unified into month by dividing by 12, and outcomes were aggregated to yearly values for final analyses.

## Prevalence

The prevalence in group $i$ in year $t$ is:

$$P_{i}(t)=\sum_{\text{j=4}}^{15} X_{i,j}(t)/\sum_{\forall j} X_{i,j}(t)$$

The prevalence of the whole MSM population in year $t$ is:

$$P(t)=\left(\sum_{\text{j=4}}^{15} X_{1,j}(t)+\sum_{\text{j=4}}^{15} X_{2,j}(t)\right)/\left(\sum_{\forall j} X_{1,j}(t)+\sum_{\forall j} X_{2,j}(t)\right)$$

## Number of new cases

The number of new cases in group $i$ in year $t$ is:

$$NI_{i}(t)=\sum_{j\geq4} \lambda_{1,j}^{i}(t)\times X_{i,1}(t)+\sum_{j\geq4} \lambda_{2,j}^{i}(t)\times X_{i,2}(t)+\sum_{j\geq4} \lambda_{3,j}^{i}(t)\times X_{i,3}(t)$$

The number of new cases of the whole MSM population in year $t$ is:

$$NI(t)=NI_{1}(t)\text{+}NI_{2}(t)$$

## Incidence rate

The incidence rate in group $i$ in year $t$ is:

$$I_{i}(t)=NI_{i}(t)/\sum_{\forall j} X_{i,j}(t)$$

The incidence rate of the whole MSM population in year $t$ is:

$$I(t)=\left(NI_{1}(t)\text{+}NI_{2}(t)\right)/\left(\sum_{\forall j} X_{1,j}(t)\text{+}\sum_{\forall j} X_{2,j}(t)\right)$$

## Time required to eliminate HIV

The time required to eliminate HIV is:

$$t_{e}=min\left\{t:I\left(t\right)<0.001\right\}-2022$$

# Model calibration

Key parameters were sampled by using a $Beta$ distribution. Here, for each key parameter, $Beta\left(2,2\right)$ was used and shifted and scaled to control the range of the possible value. Table S2 shows the list of the key parameters and their corresponding distributions.

Sensitivity analysis was conducted by randomly sampling the key parameters from their corresponding distributions simultaneously. We sampled 1000 times to obtain 1000 key parameter sets. Each set was combined with the other fixed parameters as a complete parameter set, and the model run with this complete parameter set to obtain one set of annual prevalence estimates. Ultimately, 1000 models with 1000 estimations were generated.

The estimations of the prevalence were calibrated against the prevalence from 2010 to 2016. A deviance-based loss was calculated using the following formula:

$$D_{s}=2\sum_{t=2010}^{2016} \left(P_{t}\ln\left(P_{t}/\hat{P}_{t,s}\right)-P_{t}+\hat{P}_{t,s}\right)$$

Where $D_{s}$ is the deviance of the s*th* sampling, $\hat{P}_{t,s}$is the estimated prevalence in sample $s$year $t$, and $P_{t}$ is the prevalence in year $t$. The 100 models with the lowest deviance were retained as the final model set. The weighted mean of the 100 model was the final estimation, and the range of the 100 models formed the uncertainty range.

Table S2 Sensitivity distributions of key parameters

| **Definition** | **Value** | **Range** | **Distribution** |
| --- | --- | --- | --- |
| ***Demographic*** |  |  |  |
| Initial population (age 18-59) |  |  |  |
| MSM (%) | 3.5% | 3% – 4% | 0.01*Beta(2,2)+0.03 |
| ***Behavioral*** |  |  |  |
| Annual number of partners |  |  |  |
| MSM, total | 4.1 | 3 – 5 | 2*Beta(2,2)+3 |
| High risk MSM | 14.1 | 13 – 15 | 13*Beta(2,2)+2 |
| Condom use (% of sexual encounters) |  |  |  |
| Condom use rate | 35% | 32% – 39% | [0.2*Beta(2,2)+0.9]*35% |
| Others |  |  |  |
| Proportion of members of one group having sexual interaction with members of the other group | 0.3 | 0.27 – 0.33 | [0.2*Beta(2,2)+0.9]*0.3 |
| ***Biomedical*** |  |  |  |
| HIV testing |  |  |  |
| Proportion of population tested in past 12 months, % | 35% | 32% – 39% | [0.2*Beta(2,2)+0.9]*35% |
| Monthly entry rate to ART |  |  |  |
| Acute | 0.2 | 0.18 – 0.22 | [0.2*Beta(2,2)+0.9]*0.2 |
| Asymptomatic (CD>500) | 0.29 | 0.26 – 0.32 | [0.2*Beta(2,2)+0.9]*0.29 |
| Asymptomatic (200<=CD4<=500) | 0.38 | 0.34 – 0.42 | [0.2*Beta(2,2)+0.9]*0.38 |
| AIDS (CD4<200) | 0.43 | 0.39 – 0.47 | [0.2*Beta(2,2)+0.9]*0.43 |

Table S3 The impact of PrEP at different level for each group

| **PrEP coverage** | **PYIPAI of low-risk group**  (Sensitivity range) | **PYIPAI of high-risk group**  (Sensitivity range) |
| --- | --- | --- |
| 10% | 60.49 (39.94, 148.14) | 0.82 (0.72, 1.01) |
| 20% | 57.96 (38.48, 138.77) | 0.95 (0.85, 1.15) |
| 30% | 55.97 (37.30, 131.80) | 1.04 (0.93, 1.26) |
| 40% | 54.42 (36.41, 126.62) | 1.10 (0.98, 1.35) |
| 50% | 53.22 (35.72, 122.68) | 1.15 (1.01, 1.41) |
| 60% | 52.28 (35.17, 119.63) | 1.18 (1.03, 1.46) |
| 70% | 51.52 (34.73, 117.21) | 1.20 (1.04, 1.50) |
| 80% | 50.91 (34.36, 115.24) | 1.22 (1.05, 1.54) |
| 90% | 50.39 (34.05, 113.62) | 1.23 (1.06, 1.56) |
| 100% | 49.96 (33.79, 112.26) | 1.24 (1.07, 1.58) |
| **Average PYIPAI** | **52.84 (35.50, 121.42)** | **1.14 (0.99, 1.42)** |

* PYIPAI: Person-years intervention per averted infection

Table S4 Years of elimination under different intervention scenarios

| **Intensity** | **Intervention** | **Year of HIV elimination** | | | | | | |
| --- | --- | --- | --- | --- | --- | --- | --- | --- |
|  |  | **Scenario 1**  (Sensitivity range) | **Scenario 2**  (Sensitivity range) | **Scenario 3**  (Sensitivity range) | **Scenario 4**  (Sensitivity range) | **Scenario 5**  (Sensitivity range) | **Scenario 6**  (Sensitivity range) | **Comprehensive interventions**  (Sensitivity range) |
| Weak | Partner reduction: 10% | After 2050 |  | After 2050 |  |  |  | 2044  (2043 – 2045) |
|  | Condom use rate: 40% |  | After 2050 |  |  |  |  |  |
|  | Testing and treatment: 50% |  |  |  | After 2050 |  | After 2050 |  |
|  | PrEP coverage rate: 10% |  |  |  |  | After 2050 |  |  |
| Moderate | Partner reduction: 20% | After 2050 |  | After 2050 |  |  |  | 2030  (2029 – 2031) |
|  | Condom use rate: 50% |  | After 2050 |  |  |  |  |  |
|  | Testing and treatment: 70% |  |  |  | After 2050 |  | 2038  (2035 -2039) |  |
|  | PrEP coverage rate: 20% |  |  |  |  | After 2050 |  |  |
| Strong | Partner reduction:30% | After 2050 |  | 2043  (2038 –2048) |  |  |  | 2027  (2026 – 2027) |
|  | Condom use rate: 60% |  | After 2050 |  |  |  |  |  |
|  | Testing and treatment: 90% |  |  |  | After 2050 |  | 2032  (2031 -2033) |  |
|  | PrEP coverage rate: 30% |  |  |  |  | 2047  (2044 –2050) |  |  |

* Definition of elimination: Incidence rate<**1/10000** person-years

# References

1. Li J, Peng L, Gilmour S, et al. A mathematical model of biomedical interventions for HIV prevention among men who have sex with men in China. *BMC Infect Dis* 2018; **18**(1): 600.

2. Fine PE. Herd immunity: history, theory, practice. *Epidemiol Rev* 1993; **15**(2): 265-302.

3. Bianchi A, Hillen T, Lewis MA, Yi Y. The Dynamics of Biological Systems: Springer; 2019.

4. Heffernan JM, Smith RJ, Wahl LM. Perspectives on the basic reproductive ratio. *J R Soc Interface* 2005; **2**(4): 281-93.

5. van den Driessche P, Watmough J. Reproduction numbers and sub-threshold endemic equilibria for compartmental models of disease transmission. *Math Biosci* 2002; **180**: 29-48.
